# Approach to the Management of Hypertriglyceridemia Complicated With Acute Pancreatitis in Pregnancy: A Case Report

**DOI:** 10.7759/cureus.56006

**Published:** 2024-03-12

**Authors:** Hamad S Saif, Basma Al-Ansari, Gulmeen Raza, Maha Ghorabah

**Affiliations:** 1 General Practice, King Hamad University Hospital, Busaiteen, BHR; 2 Obstetrics and Gynecology, King Hamad University Hospital, Busaiteen, BHR

**Keywords:** pancreatitis, severe hypertriglyceridemia, management, gynaecology and obstetrics, gastroenterology, endocrinology, hematology, multi-disciplinary team approach, pregnancy, hypertriglyceridemia-induced acute pancreatitis

## Abstract

This is a case of a 32-year-old woman, Gravida 3 para 2, previous two cesarean sections, who presented to our emergency department at 24+3 weeks of gestation complaining of severe epigastric pain radiating to the back. She was diagnosed with severe hypertriglyceridemia complicated with acute pancreatitis and was managed by a multi-disciplinary team, which included obstetrics, gastroenterology, endocrinology, hematology, nutrition, and ICU team. Initially, conservative treatment was employed for her management. She was placed on nil per oral status and initiated on a normal saline infusion at a rate of 150 ml/hour, along with insulin infusion at 0.1 unit/kg/hour and dextrose (D5) at 80 ml/hour. Additionally, she received omeprazole, meropenem, clexane (40 mg once daily subcutaneous injection), iron, vitamin supplements, and analgesics as required. Subsequently, due to the failure of the initial conservative medical management, the patient was admitted to the ICU. Plasmapheresis was performed after the insertion of a vascath, using 3000 ml of albumin 5% as replacement fluid and oral calcium. Following this, she was prescribed Omacor (Omega 3) at a dosage of 2 grams orally twice daily, along with a low carbohydrate and fat diet, to manage her triglyceride levels. After the removal of the central line, her triglycerides increased to 14.3 mmol/L, leading to the initiation of fenofibrate at a daily dose of one tablet. With persistent elevation to 16.4 mmol/L, Lipitor at 40 mg once daily was introduced. Following this intervention, her triglyceride levels stabilized, and her overall condition improved. She was discharged at 25+1 weeks with a prescribed regimen, and scheduled follow-ups were arranged in the endocrine and obstetrics clinics. At 36 weeks of gestation, she presented to the emergency room with abdominal, back, and leg pain. Fetal distress, indicated by fetal tachycardia (170-180 bpm) on cardiotocography, prompted an urgent category 1 cesarean section, which proceeded without complications.

## Introduction

Lipids comprise any class of organic complexes that are difficult to dissolve in water but are soluble in organic solvents. Lipid metabolism undergoes a few physiological changes during pregnancy, which are necessary for healthy fetal development but result in alterations in lipid levels [1]. Triglyceride (TG) levels increase by a factor of more than three from 12 weeks of gestation to the end of the third trimester to support energy storage in the mother and promote embryonic development [2]. TG levels below 1.7 mmol/L (150 mg/dL) are considered normal. In the last few weeks of pregnancy however, it is possible for the TG level to surpass 11.3 mmol/L (1,000 mg/dL), which is considered severe hypertriglyceridemia [3]. Severe hypertriglyceridemia during pregnancy can result in many complications including acute pancreatitis (AP), hyper-viscosity syndrome, pre-eclampsia, macrosomia, premature birth, or fetal mortality [4]. AP is an uncommon yet potentially fatal condition that affects both the mother and neonate. Its incidence is estimated to be between 3 and 10 per 10,000 live births. In contrast to other etiologies, AP induced by hypertriglyceridemia during pregnancy exhibits a higher propensity for severe manifestation, correlates with an unfavorable prognosis, and augments both maternal and neonatal mortality [5]. This case report will focus on the management of hypertriglyceridemia during pregnancy in the context of a pregnant woman who developed AP-complicated hypertriglyceridemia in our hospital.

## Case presentation

A 32-year-old Indian woman, Gravida 3 para 2, previous two cesarean sections, at 24+3 weeks of gestation, presented to the emergency department in November 2022 complaining of severe epigastric pain radiating to the back. Her previous pregnancy was terminated at 32 weeks of gestation by emergency cesarean section in 2019 due to AP secondary to hypertriglyceridemia. She was admitted to the ICU at that time due to tachypnea and tachycardia and was started on an insulin infusion, and then the decision to terminate was taken in view of her unstable condition. She was switched to oral medications after delivery. The patient had no other relevant medical history; she had normal TG levels outside pregnancy, her family health history was irrelevant, and she had no clinical stigmata associated with hypertriglyceridemia on physical examination, was non-alcoholic, and was a non-smoker. 

Her laboratory results shown in Table 1 indicate that there is an elevation in lipase, amylase, cholesterol, TGs, very low-density lipoprotein (VLDL) cholesterol, total cholesterol/high-density lipoprotein (HDL) ratio, and C-reactive protein (CRP).

**Table 1:** Laboratory tests on hospital admission LDL: Low-density lipoprotein; HDL: high-density lipoprotein; VLDL: very low-density lipoprotein; CRP: C-reactive protein

Laboratory Test	Results	Normal range
Lipase	268 U/L	12-53 U/L
Amylase	270 U/L	30-180 U/L
Cholesterol	16.7 mmol/L	3.6-5.2 mmol/L
Triglycerides	49.1 mmol/L	0-1.7 mmol/L
LDL Cholesterol	1.92 mmol/L	1.6-4.7 mmol/L
HDL Cholesterol	1.6 mmol/L	0.83-1.86 mmol/L
VLDL Cholesterol	10.26 mmol/L	0.1-1.7 mmol/L
Total Cholesterol/HDL ratio	9.5	0-5
Calcium	1.72 mmol/L	2.12-2.62 mmol/L
Albumin	29.8 g/L	38-50 g/L
CRP	109.8 mg/L	0-10 mg/L

**Figure 1:**
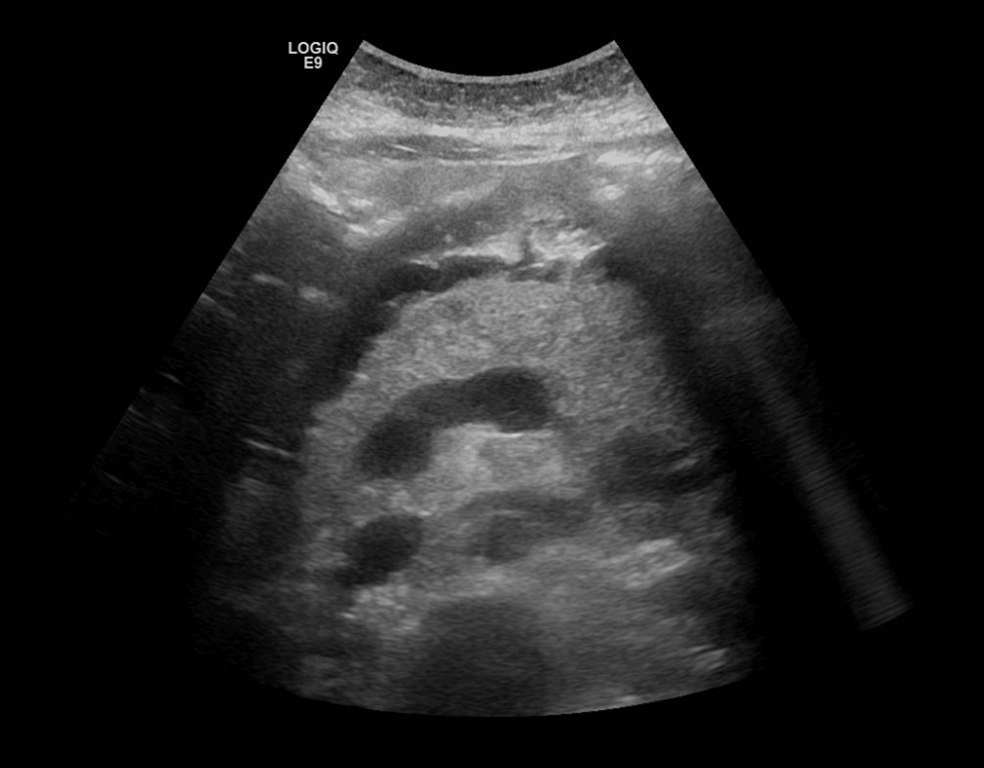
Ultrasound abdomen showing diffusely enlarged pancreas more at the tail with an irregular surface and surrounding peri-pancreatic free fluid suggestive of acute pancreatitis

**Figure 2:**
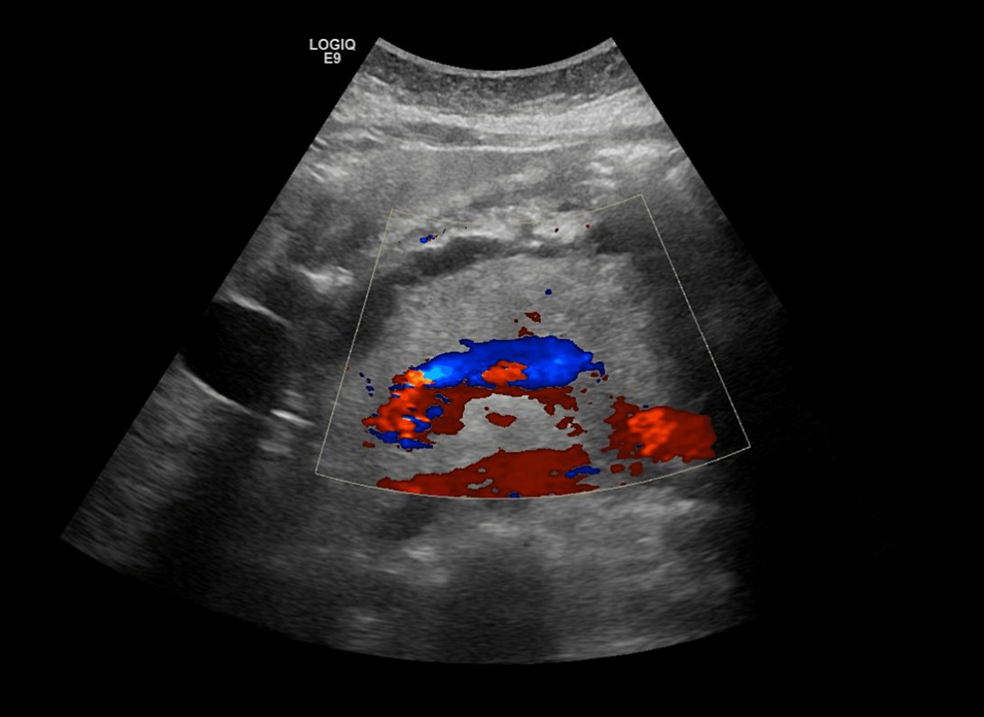
Doppler ultrasound abdomen showed diffusely enlarged pancreas more at the tail with an irregular surface and surrounding peri-pancreatic free fluid suggestive of acute pancreatitis

Abdominal ultrasound showed diffusely enlarged pancreas more at the tail with an irregular surface and surrounding peri-pancreatic free fluid suggestive of AP (Figures 1, 2). Minimally dilated intra-hepatic biliary radicles, with a normal diameter of the CBD (around 0.5 cm), enlarged fatty liver, no evidence of gallstones, mild ascitic free fluid in the abdomen, and viable intrauterine fetus were seen.

She was admitted to the antenatal ward with an impression of hypertriglyceridemia-induced AP. A multidisciplinary team was involved in her management plan including obstetrics, gastroenterology, endocrinology, hematology, nutrition, and ICU team.

She was initially kept nil per oral (NPO) and was started on normal saline 150 ml/ hour insulin infusion 0.1 unit/kg/hour and dextrose (D5) 80ml/hour with monitoring of her random blood sugar hourly with dextrose (D5) adjustment accordingly. Also, she received omeprazole, meropenem, clexane 40 mg once daily subcutaneous injection, iron, vitamin supplements, and analgesics when needed.

After initial conservative medical management failed, the patient was admitted to the ICU and underwent plasmapheresis after insertion of a vascath with 3000 ml of albumin 5 % as replacement fluid and oral calcium. Following that her TG improved (from 49.1 to 5.1 mmol/L) due to plasmapheresis, insulin, and being NPO.

On Day 2, the patient was started on Omacor (Omega 3) 2 grams orally twice daily to maintain her triglycerides at the target level (<5.6 mmol/L) once she started feeding orally. On Day 3, the patient was started on a low-carb and low-fat diet.

On Day 4, her TG levels exhibited a trend toward 4.3 mmol/L, and her central line was removed. On repeating her labs, her TGs raised back to 14.3 mmol/L, so she was started on fenofibrate 145 mg once daily.

The following day, her TGs were elevated to 16.4 mmol/L, even though she was on fenofibrate, so she was started on Lipitor 40 mg once daily. Her TG levels stabilized after that and her condition improved so she was discharged on this regimen at 25+1 weeks, with regular follow-ups with lab results in the endocrine and obstetrics clinics.

The patient presented to the obstetrics and gynecology emergency room at 36 weeks of gestation with complaints of lower abdominal pain, back pain, and leg pain in the last 24 hours. Her cardiotocography showed fetal tachycardia with a heart rate of 170-180 beats per minute. Therefore, the patient was taken for category 1 cesarean section in view of fetal distress which went uneventful. She was discharged in stable condition on a home regimen of Lipitor 40 mg at bedtime, fenofibrate 145 mg at bedtime, and Omacor (Omega 3) two tablets twice daily orally. The patient was also advised to take clexane injections for 10 days, recommended lifestyle modifications, to maintain a low-salt and low-fat diet, and to exercise as tolerated. She was given a six-week follow-up in the gynecology clinic but failed to attend the appointment and subsequently did not contact us to reschedule.

## Discussion

Hypertriglyceridemia in pregnancy is the third most prevalent cause of AP accounting for up to 14.4%. Other causes include gallstones and alcohol consumption comprising 65% and 5-10% of the cases respectively [6].

Hypertriglyceridemia can manifest in two ways: primary, as in the case of congenital chylomicronemia syndrome, which is caused by a deficiency of apoproteins C-II or lipoprotein lipase, or secondary to metabolic syndrome, obesity, undiagnosed or uncontrolled diabetes mellitus, alcohol consumption, drug use (including tamoxifen, steroids, diuretics, beta-blockers, and atypical antipsychotics), or absence of any predisposing factor [6].

During pregnancy, estrogen causes changes in lipid metabolism to provide adequate nutrition for the fetus. Some women with a genetic predisposition or an additional triggering condition may experience severe gestational hypertriglyceridemia. It can lead to numerous life-threatening consequences, such as AP [7].

There is still a lack of clarity on the pathophysiological mechanism of AP caused by hypertriglyceridemia [8]. One theory suggests that TG-rich lipoproteins stimulate lipolysis leading to elevated levels of free fatty acids (FFAs). This, in turn, causes damage to the acinar cells and vascular endothelium of the pancreas. Moreover, in laboratory investigations, it was discovered that fatty acids cause damage to the mitochondria as a result of high levels of TGs in the blood. The ischemia-induced acidic environment exacerbates the toxicity of the FFAs leading to heightened pancreatic damage [7].

The International Association of Pancreatology and the American Pancreatic Association have stipulated that the diagnosis of AP requires the fulfillment of at least two out of the three specified criteria. The criteria for diagnosis include clinical symptoms of upper abdomen discomfort, laboratory findings of serum amylase or lipase levels above three times the upper limits of normal, and indicative imaging results [9]. In reference to our case report, our patient fulfilled all three requirements for the diagnosis of AP.

Treating severe gestational hypertriglyceridemia is a complicated process. Due to its rarity, there are currently no documented guidelines available for managing this illness. An interdisciplinary strategy is required, involving collaboration among obstetrics, endocrinology, metabolic medicine, and dietitians. This is acknowledged as the cornerstone of effective management [10].

The proper diet and use of nutritional supplements are crucial for keeping TG levels within the normal range. Patients should be instructed to have a diet that is both isocaloric and low in fat, with less than 20% of total calories derived from fat. Omega 3 fatty acids, which consist of both eicosapentaenoic acid and docosahexaenoic acid, are widely regarded as the fundamental component of nutritional supplement therapy. The compounds decrease hepatic lipogenesis and promote fatty acid oxidation in the liver and skeletal muscle. There have been studies that showed a decrease in TG levels by 25 to 30% following the commencement of this treatment [11].

In cases of severe complicated hypertriglyceridemia not controlled by diet and nutritional supplements, patients may require hospitalization, to be kept nil per mouth, and to be started on 5% intravenous dextrose and insulin to optimize their TG levels [12]. 

There are currently no guidelines for hypertriglyceridemia drug prescriptions during pregnancy, and most treatments are based on case reports with variable degrees of success which include statins, niacin, and fibrates. Although statin studies are disputed, statin use among pregnant women is restricted because there may be some potential teratogenic consequences [13]. Niacin is considered in pregnancy as a category C drug which has been shown to boost HDL levels while decreasing LDL levels. However, its use is restricted due to its significant side effects, which include hot flushes, gastrointestinal upset, and potentially liver damage [12,13]. During pregnancy, fenofibrate is classified as category C although, some case reports indicated that it was safe to use during pregnancy. This medication lowers plasma TG levels by 50% while increasing HDL cholesterol by 20%. It also reduces very low-density lipoprotein secretion and increases plasma TG lipolysis [8].

Another treatment modality that has been suggested is intravenous heparin. It functions through the activation of lipoprotein lipase. Although it has shown efficacy in non-pregnant individuals, its usage during pregnancy may be linked to hemorrhage-related complications and could exacerbate cases of pancreatitis [1].

Plasmapheresis may be explored for patients suffering from severe hypertriglyceridemia that does not respond to other treatment methods as it dramatically lowers TG levels [1,13]. However, its high cost, thrombosis hazards, biological availability, and catheter-associated inflammation have all limited its broad use [13].

In the case of our patient, a multidisciplinary team consisting of obstetrics, gastroenterology, endocrinology, hematology, dietitians, and ICU team were involved in her management plan. She was initially started on conservative management by being kept NPO and was started on intravenous fluids, intravenous insulin infusion, and dextrose 5% intravenous. After conservative management failed, plasmapheresis was started which helped lower her TG levels. However, only after starting her on Omega 3 supplements, fenofibrate, and statins, her TGs return to normal. Neither niacin nor IV heparin was used in her case. However, she was kept on heparin (clexane) intramuscular during her hospital stay and was discharged on it.

## Conclusions

To conclude, gestational hypertriglyceridemia complicated with AP is a rare condition with no documented guidelines to date. Therefore, its management requires the cooperation of a multi-disciplinary team consisting of obstetricians, endocrinologists, gastroenterologists, and dietitians. Hospital admission is necessary when TG levels are uncontrolled with diet and medications. Some medications used in the treatment are categorized as category C which should be used only when the benefits outweigh the risks. Additional studies should be conducted to provide a standardized set of guidelines regarding this illness for clinicians to adhere to in the future.
